# Decentralized databases in biomedical research: lessons from recent events: The recent shutdown of critical health databases by the US CDC is a wake-up call for the research community about the vulnerability of centralised databases

**DOI:** 10.1038/s44319-025-00417-5

**Published:** 2025-03-18

**Authors:** Alfonso Valencia

**Affiliations:** ICREA and Life Sciences Department, Barcelona Supercomputing Center (BSC-CNS), Barcelona, Spain

**Keywords:** Computational Biology, Economics, Law & Politics, Methods & Resources

## Abstract

Biological and medical databases are crucial resources without which biomedical research would come to a halt. However, centralized databases often face vulnerabilities, particularly when dependent on single institutions. In contrast, distributed databases enhance resilience, encourage international collaboration, and promote scientific advancement by supporting shared responsibility and improved accessibility.

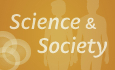

The recent actions of the US Centers for Disease Control and Prevention (CDC), in temporarily withdrawing certain online resources and reducing staff in response to presidential decrees, constitute a significant event with far-reaching implications. This episode serves as a cautionary tale, highlighting the inherent risks associated with concentrating critical scientific and medical information within a single, potentially vulnerable entity subject to political dynamics. While centralized resources might be attractive owing to their simplicity, they are also susceptible to political, natural, and technical threats. This underscores the urgent need for decentralized databases and resources to safeguard against such disruptions.

While centralized resources might be attractive owing to their simplicity, they are also susceptible to political, natural, and technical threats.

## The importance of biological databases

Biological databases are the backbone of scientific progress. Pretty much everything in biology and biomedicine, from genomics and drug development to public health, relies on the data stored therein. Databases are not just storage units; they are curated, organized resources that allow researchers to access, analyse and build on existing knowledge. Take, for example, the Protein Data Bank (PDB). This global effort, with nodes in the USA, Europe, and Japan, houses tens of thousands of protein structures, and without it, breakthroughs such as AlphaFold—which won the 2024 Nobel Prize in Chemistry—wouldn’t have been possible. These databases are also essential for education, collaboration, and innovation across the life sciences. Without them, progress in medicine, agriculture, and biotechnology would slow down or simply become impossible.

… during the COVID-19 pandemic, the CDC’s data on case counts, hospitalizations and deaths was critical for tracking the spread of the virus.

The CDC’s medical repositories play an analogous role in public health by storing critical health data: disease surveillance records, vaccination rates, outbreak reports, public health guidelines, and more. This data is essential for researchers and public health officials trying to track diseases, develop prevention strategies, and shape policies (Box 1). For instance, during the COVID-19 pandemic, the CDC’s data on case counts, hospitalizations, and deaths was critical for tracking the spread of the virus. Similarly, CDC data on smoking rates and tobacco-related illnesses has been instrumental in shaping anti-smoking policies. These examples show how these repositories are a powerful tool for improving public health, guiding policy, and saving lives. They also play a key role in women’s health and health equality. Without these resources, we’d be flying blind when it comes to understanding diseases, developing vaccines, and tackling global health crises. Just like the PDB revolutionized structural biology, the CDC’s databases are indispensable for advancing public health.

Box 1. CDC resources

1. Tracking and Responding to Disease Outbreaks: During the COVID-19 pandemic, the CDC's data on case counts, hospitalizations, and deaths was critical for tracking the spread of the virus.
2. Vaccine Development and Distribution: CDC data on vaccine effectiveness and side effects was used to monitor the rollout of COVID-19 vaccines.
3. Public Health Policy and Guidelines: CDC data on smoking rates and tobacco-related illnesses has been instrumental in shaping anti-smoking campaigns.
4. Monitoring Health Disparities: CDC data on HIV infection rates has highlighted disparities among different populations.
5. Environmental Health and Safety: CDC data on lead levels in children has been used to identify communities at risk of lead exposure.

Examples of CDC Data in Women's Health and Health Equality

1. Maternal Mortality and Pregnancy-Related Complications: CDC data on maternal mortality has revealed stark racial disparities.
2. Reproductive Health and Family Planning: CDC data on contraceptive use and access has informed programs to improve family planning services.
3. Breast and Cervical Cancer Screening: CDC data on cancer screening rates has been critical for programs providing free or low-cost mammograms.
4. Violence Against Women: CDC data on intimate partner violence has highlighted the prevalence of physical and sexual violence against women.
5. Chronic Diseases in Women: CDC data on heart disease has shown that it is the leading cause of death for women in the USA.

## The risks of centralized resources

Centralized databases have intrinsic advantages: they are easy to establish and maintain and depend on a small number of decision bodies. They have also critical vulnerabilities, however. Among these are: Significant implicit computational cost, for example, for backups and data preservation that depend on a single institution. In contrast, decentralized databases compensate part of these costs. Centralised databases may have more difficulties in recruiting and offering opportunities for professional development. They are potentially vulnerable to natural disasters such as earthquakes, floods or hurricanes. Similarly, central systems can be affected by technical failures, such as server crashes, cyberattacks or software bugs. These failures can lead to data loss or prolonged downtime, disrupting research and public health efforts. The dependency on a single institution or organization, might create difficulties in dealing with fluctuations in funding, that could be better tempered by well-organized collaboration and sharing of resources. Often, obtaining funding from additional sources is easier if a database is already funded by other institutions. As we see now, centralised databases can be subject to political interference, including censorship, manipulation or even shutdowns.

These vulnerabilities underscore the urgent need for decentralized databases and repositories to safeguard against disruptions. Decentralized systems distribute data across multiple nodes, thereby offering enhanced resilience. They also align with the principles of open science by ensuring broader access to research findings. Indeed, being shared between different groups and institutions is an important criteria for the evaluation of core and national resources by the European Infrastructure on Life Sciences- ELIXIR (www.elixir.eu).

As we see now, centralised databases can be subject to political interference, including censorship, manipulation or even shutdowns.

## Decentralized resources and federated databases

Thus, decentralized resources are crucial for ensuring data resilience, accessibility, and collaboration in biological and biomedical research by reducing risks associated with centralized systems. There are various types of decentralized resources.

Full Data Duplication: In this model, data is fully replicated across multiple nodes to ensure redundancy and resilience. An example is the European Genome-Phenome Archive (EGA, https://ega-archive.org/), which stores copies of genomic data at two different locations. This approach guarantees data availability even if one node fails but it requires significant storage capacity and maintenance.

Building on Existing Resources: These systems enhance and aggregate data from existing sources without duplicating all the information. For instance, Europe PMC (https://europepmc.org) builds on PubMed Central (PMC https://pmc.ncbi.nlm.nih.gov) and integrates literature and additional data from multiple sources, providing a unified platform for researchers. This model improves interoperability and functionality but relies on an external data source.

… decentralized resources are crucial for ensuring data resilience, accessibility, and collaboration in biological and biomedical research by reducing risks associated with centralized systems.

Distributed Workload: Tasks such as data curation and annotation are shared among multiple institutions without duplication. UniProt is an example, where partners collaborate to maintain a protein database, each contributing their specific expertise. This approach optimizes efficiency and collaboration but requires efficient coordination.

Federated databases: In this case, different entities maintain their own data but they use the same infrastructure to make the data interoperable at the syntactic and semantic level. It enables common access and operation by running the same software in the independent sites, fulfilling the federated access and federate analysis paradigm.

These categories should not be seen as completely independent, and a number of databases are organized in collaboration between institutions in different countries with different models of association (Box 2). A relevant example is the European Genome-Phenome Archive (EGA), a decentralized system run by EMBL-EBI (https://www.ebi.ac.uk) and Centre for Genomic Regulation (CRG https://www.crg.eu), with data stored at both EBI and the Barcelona Supercomputing Center (BSC-CNS) (https://www.bsc.es). Interesting, EGA can also adapt to operate as a federated system (federated EGA or fEGA: https://ega-archive.org/about/projects-and-funders/federated-ega/), allowing genomic and phenotypic data to be stored and accessed across multiple countries without moving them from their legal sites, while keeping the data secure and private.

While decentralized systems offer many advantages, they also come with challenges. Distributed databases require a different mechanism to support various hubs. This could involve international agreements, the organization of consortia or public-private partnerships to ensure sustainable funding. In a distributed system, the question of who controls the data and ensures its curation and organization becomes critical, requiring adequate governance frameworks to ensure that data remains open and accessible. Creating independent databases based on other existing systems, such as the PMC/EuroPMC, requires additional funding. However, the cost of not having redundancy could be far greater in the event of data loss or disruption. Investment in mirroring and duplication is essential to safeguard data, to maintain and replace backups, ensure data resilience and long-term accessibility.

Decentralization in the case of highly confidential data, including genomics and medical data, have additional security requirements. Current developments in computational protocols and cryptography, including Secure Multi-Party Computation, blockchain technology, homomorphic encryption or differential privacy, and the availability of federated access protocols (FLOWER, TensorFlow Federated, PySyft, OpenFL, FATE Federated AI Technology Enabler) now make it easier to share data securely. These technologies are already being explored in biomedical research. For instance, the GA4GH has developed a framework for responsible sharing of genomic and health-related data (https://www.ga4gh.org/framework/) and the EUCAIM (https://cancerimage.eu) project has developed a system based on FLOWER for secure sharing of medical imaging data.

Box 2. Examples of biological databases shared between institutions Protein Data Bank (PDB): A global repository for 3D structural data of proteins, nucleic acids, and complex assemblies. It's a collaborative effort involving: RCSB PDB (USA), hosted by Rutgers University and UC San Diego; PDB Europe (PDBe), managed by the European Bioinformatics Institute (EBI); and PDB Japan (PDBj), operated by Osaka University.

UniProt: A comprehensive resource for protein sequence and functional info. It's a collaboration between the EBI, Protein Information Resource (PIR) based at Georgetown University in the USA and the Swiss Institute of Bioinformatics (SIB).

GenBank and European Nucleotide Archive (ENA) are managed by the National Center for Biotechnology Information (NCBI) in the USA and the European Nucleotide Archive (ENA) hosted by the EBI.

European Genome-Phenome Archive (EGA) and federated EGA (fEGA). EMBL-EBI and CRG manage EGA, with data stored at both EBI and the Barcelona Supercomputing Center (BSC-CNS). Federated EGA includes nodes in Finland, Germany, Norway, Spain, Sweden, Poland, and Portugal. (https://ega-archive.org/about/projects-and-funders/federated-ega/)

PMC (PubMed Central) including PubMed, MEDLINE and NCBI Bookshelf, as well as Europe PMC, are repositories for life-sciences literature. PMC is maintained by the US National Library of Medicine (NLM) while Europe PMC is hosted by the EMBL-EBI.

These databases and others provide researchers with the tools and data needed to drive innovation in biology and medicine. By sticking to standardized formats, interoperability frameworks and open-access principles, they ensure that scientific knowledge remains a shared global resource.

## The fragility of biological databases

Biological databases are the cornerstone of modern biomedical research, serving specific purposes from genomic sequences to protein structures and clinical data, yet they are inherently fragile. The journal *Nucleic Acids Research* publishes an annual special issue dedicated to describing new and updated databases, highlighting the sheer volume and diversity of these resources. ELIXIR compiles a large collection of databases provided by its national nodes, offering researchers access to a wide range of biological data and tools, and has developed an evaluation system to select “core databases” (https://elixir-europe.org/platforms/data/core-data-resources). Similarly, the Global Biodata Coalition (GBC https://globalbiodata.org) identifies essential resources at a global scale. These resources and their continued operation are vital for the biological and biomedical sciences.

These databases require skilled professionals to manage, curate and update them, and specialized infrastructure and continuous funding to maintain them. However, many are underfunded or rely on indirect funding sources, making them vulnerable to disruptions. Additionally, the fragility of databases is exacerbated by the increasing pressure of artificial intelligence (AI), such as the EU’s AI Factories (https://ec.europa.eu/commission/presscorner/detail/en/ip_24_6302), which aim to provide cutting-edge computational power and AI technologies, demanding accessible high-quality data for training and evaluating models, a demand for which the databases will require additional resources.

The recent events in the USA are therefore a wake-up call for the scientific community. We cannot take the accessibility and integrity of biomedical data for granted. By embracing decentralized systems, we can protect critical information from political interference, natural disasters, and technical failures. The time for action is now, before the next crisis necessitates a far more extensive and costly response. We need to ensure that the pursuit of knowledge stays free, open, and resilient, no matter what.

By embracing decentralized systems, we can protect critical information from political interference, natural disasters, and technical failures.

## Supplementary information

Peer Review File

