# Supplementary material for: Decentralized databases in biomedical research: lessons from recent events: The recent shutdown of critical health databases by the US CDC is a wake-up call for the research community about the vulnerability of centralised databases
Source: EMBO Rep. 2025 Mar 18;26(7):1679–81. doi: 10.1038/s44319-025-00417-5 (PMC11977236; doi:10.1038/s44319-025-00417-5)
Supplement: Supplementary file 1 — Peer Review File [file 44319_2025_417_MOESM1_ESM.pdf]

EMBO reports

Decentralized Databases in Biomedical Research: Lessons from Recent Events

Alfonso Valencia

Corresponding author(s): Alfonso Valencia (alfonso.valencia@bsc.es)

Review Timeline:

Submission Date:	27th Feb 25
Accepted:	4th Mar 25

Editor: Holger Breithaupt

Transaction Report: This article was editorially reviewed.
